# Tourniquet use in routine primary total knee arthroplasty is associated with a higher transfusion rate and longer postoperative length of stay: a real-world study

**DOI:** 10.1186/s12891-020-03623-5

**Published:** 2020-09-18

**Authors:** Hong Xu, Jingli Yang, Jinwei Xie, Zeyu Huang, Qiang Huang, Guorui Cao, Fuxing Pei

**Affiliations:** 1 Department of Orthopaedic surgery and National Clinical Research Center for Geriatrics, West China Hospital, Sichuan University, No.37, Guoxue Road, Wuhou district, Chengdu, 610041 China; 2 College of Earth and Environmental Sciences, School of Public Health, Lanzhou University, No.222, Tian Shui Nan Lu Road, Chengguan district, Lanzhou, 730000 Gansu China; 3 Department of Orthopaedic surgery, West China Hospital, Sichuan University, No.37, Guoxue Road, Wuhou district, Chengdu, 610041 China

**Keywords:** Tourniquet, Knee, Arthroplasty, Transfusion, Length of stay

## Abstract

**Background:**

In an enhanced recovery after surgery program, a growing number of orthopedists are reconsidering the necessity of tourniquet use in total knee arthroplasty (TKA). However, the impact of tourniquet use on transfusion rate and postoperative length of stay (PLOS) in TKA remains controversial. Therefore, we carried out a study to investigate the effect of tourniquet application in routine primary TKA on transfusion rate and PLOS.

**Methods:**

We analyzed data from 6325 patients who underwent primary unilateral TKA and divided them into two groups according to whether a tourniquet was applied during the procedure, and a tourniquet was used in 4902 and not used in 1423. The information for transfusion and PLOS was extracted from patients’ electronic health records, and the data were analyzed with logistic and linear regression analyses.

**Results:**

Following TKA, the transfusion rate and PLOS were 14.52% and 7.72 ± 3.54 days, respectively, in the tourniquet group, and 6.47% and 6.44 ± 3.48 days, respectively, in the no-tourniquet group. After adjusting for the different related variables, tourniquet use was significantly correlated with a higher transfusion rate (risk ratio = 1.888, 95% confidence interval (CI) 1.449–2.461, *P* < 0.001) and a longer PLOS (partial regression coefficient (*B*) = 0.923, 95%CI 0.690–1.156, *P* < 0.001).

**Conclusions:**

Our findings suggested that tourniquet use in routine primary TKA was related to a higher transfusion rate and a longer PLOS. The impact of tourniquet use on transfusion rate and PLOS should be taken into account in clinical practice.

Total knee arthroplasty (TKA) is considered one of the most successful orthopedic surgical treatments for end-stage knee diseases because it can relieve pain, improve lower limb function, and improve patients’ quality of life [1, 2]. Tourniquets have been used in TKA since the procedure was first introduced [3, 4]. Until 2011, tourniquets were used in approximately 90% of the 12,048 primary TKAs performed in Sweden [5]. Proponents of tourniquet use in TKA believe that it can reduce intraoperative bleeding volume (which potentially offers a relatively bloodless operating field), decreases the operative time, and, perhaps most importantly, ensures a better cementation result that leads to effective and long-term implant fixation [6, 7]. However, opponents of tourniquets propose that their use in TKA can lead to skin blistering, soft-tissue damage, muscle atrophy, ischemia–reperfusion injury (which contributes to postoperative pain), hematoma at the thigh, and weakness of the quadriceps; moreover, tourniquet use can hamper early mobilization and slow down postoperative rehabilitation [8–10].

In addition, the development of enhanced recovery after surgery (ERAS) programs, especially the extensive use of tranexamic acid (TXA) (an effective and safe antifibrinolytic drug) in the perioperative period and a controlled hypotension technique during the procedure, has significantly decreased both total blood loss and intraoperative bleeding volume, and offers a blood-free operating field in TKA [11–13]. Hence, a growing number of orthopedists are questioning the validity of tourniquet use in TKA [5, 14].

Although numerous studies have assessed the impact of tourniquet use on transfusion rate and postoperative length of stay (PLOS) in TKA, tourniquet application remains controversial because most of the current literature includes limited sample sizes and wide diversity, often producing inconsistent conclusions [5, 15, 16]. Therefore, we conducted a retrospective cohort study based on a large dataset to identify (1) whether tourniquet use affect the postoperative transfusion rate, and (2) whether tourniquet use is correlated with longer PLOS after routine primary TKA (simple cases without severe varus or valgus deformity, that means the hip-knee-ankle angle < 165° or > 195°).

## Materials and methods

### Data source

A prospective multicenter study, which was sponsored by the Ministry of Health of the People’s Republic of China (project number: 201302007) and enrolled in 26 university teaching hospitals in China (included 10 national and 16 regional hospitals), was carried out to assess the efficacy and safety of perioperative management measure and improve the development enhance recovery after surgery of TKA and total hip arthroplasty in China from 2013 to 2016. The large database generated from the study was analyzed secondarily for evaluating the effect of tourniquet use in TKA on transfusion rate and PLOS. Whether to use a tourniquet during surgical procedure in TKA was determined by the leader of each medical team. The transfusion criteria of allogeneic blood for each patient followed the specification for clinical transfusion technology issued by Ministry of Health of the People’s Republic of China in June 2000. Those were (1) hemoglobin level < 70 g/L and (2) according to the anemia degree of the patient, cardiopulmonary compensatory function, whether or not metabolic rate increased and age and other factors when hemoglobin level between 70 and 100 g/L. In addition, the PLOS was defined as the date of discharge minus the date of surgery. The database analyzed in this study included patient-level hospital discharge data provided by enrolled 26 hospitals. The accuracy and completeness of the data were inspected and verified carefully by comparison with information from hospital electronic record. This study was deemed exempt by the hospital’s institutional review board. And all patients gave their informed consent.

### Study population

The Clinical Modification procedure codes of the International Classification of Diseases, Tenth Revision (IDC - 10) was used to identified the patients who underwent TKA from the large database (*n* = 7789). Patients who had undergone revision hip and/or knee arthroplasty or bilateral TKA, with metastatic and/or bone cancer, with lower-extremity fractures, younger than 18 years, with bleeding diathesis, and received an autologous blood transfusion rather than an allogeneic blood product were excluded according to their electronic medical records (*n* = 1464). Finally, 6325 patients were included in this study, including 4902 (77.50%) patients for whom a tourniquet was used during the TKA procedure and 1423 (22.50%) patients who did not have a tourniquet applied. Tourniquet was used before skin incision for 4531 patients, while before placing the prosthesis for the remaining 371 patients in tourniquet use group. And the pressure of tourniquet was determined by the leader of each medical team. All the operations were performed under the supervision of the medical team leaders, chief surgeons or associate chief surgeons come from Grade-A Tertiary Hospital. The demographic characteristics, which including age, sex, body mass index (BMI), and diagnoses, comorbidities which including hypertension, diabetes, coronary heart disease, and chronic obstructive pulmonary diseases, preoperative drug use, results of preoperative laboratory examination, operative variables, such as anesthesia methods, American Society of Anesthesiologists (ASA) class, TXA use applicated intravenously or intravenously combined with locally, drain use, intraoperative blood loss and operative time of the included patients who underwent primary unilateral TKA were enrolled and analyzed, and all subjects also were shown in Table 1. The total dose of TXA was 1-3 g for almost all of the included patients who received TXA. The drainage tubes were removed on the first day postoperatively. The first discharge diagnoses of the included patients were classified into two types: (1) degenerative arthritis (osteoarthritis) and (2) inflammatory arthritis, which consisted of rheumatoid arthritis and ankylosing spondylitis. The ASA classification of each patient evaluated by anesthetist before surgery was divided into two class: < 3 or class ≥3. In addition, the anesthesia methods were divided into two types: general or regional anesthesia.

**Table 1:** The baseline characteristics of the patients underwent routine primary TKA

Baseline Characteristics	No Tourniquet (*n* = 1423)	Tourniquet use (*n* = 4902)	All patients (*n* = 6325)	*P* values
Demographic characteristics				
Age (M ± SD)	66.67 ± 8.77	66.58 ± 8.74	66.60 ± 8.75	0.722
Female, N (%)	1099 (77.23%)	3872 (78.99%)	4971 (78.59%)	0.155
BMI	24.89 ± 3.56	25.83 ± 3.5.36	25.62 ± 5.02	0.000*
Diagnoses, N (%)				0.145
OA	1321 (92.83%)	4603 (93.90%)	5924 (93.66%)	
Inflammatory arthritis	102 (7.17%)	299 (6.10%)	401 (6.34%)	
Comorbidities, N (%)				
Hypertension	224 (15.74%)	1483 (30.25%)	1707 (26.99%)	0.000*
Type 2 diabetes	51 (3.58%)	382 (7.79%)	433 (6.85%)	0.000*
CHD	15 (1.05%)	202 (4.13%)	217 (3.43%)	0.000*
COPD	4 (0.28%)	30 (0.61%)	34 (0.54%)	0.133
Preoperative analgesic use N (%)	241 (16.94%)	1551 (31.64%)	1792 (28.33%)	0.000*
Preoperative laboratories				
Preoperative Hb (g/L)	128.54 ± 15.24	132.04 ± 16.02	131.25 ± 15.91	0.000*
Preoperative ALB (g/L)	41.72 ± 2.54	40.86 ± 2.98	40.83 ± 2.89	0.095
Operative variables				
Anesthesia, N (%)				0.000*
General	1271 (89.32%)	3056 (62.34%)	4327 (68.41%)	
Regional	152 (10.68%)	1846 (37.66%)	1998 (31.59%)	
ASA class, N (%)				0.007*
< 3	1303 (91.57%)	4367 (89.09%)	5670 (89.64%)	
≥ 3	120 (8.43%)	535 (10.91%)	655 (10.36%)	
TXA use N (%)	639 (44.91%)	2758 (56.26%)	3397 (53.71%)	0.000*
Drain use, N (%)	564 (39.63%)	3976 (81.11%)	4540 (71.78%)	0.000*
Intraoperative blood loss (mL)	168.43 ± 100.56	166.55 ± 97.71	166.97 ± 98.36	0.526
Operative time (min)	81.60 ± 27.28	87.32 ± 29.51	86.03 ± 29.12	0.000*

*BMI* body mass index; *OA* osteoarthritis; *CHD* coronary heart disease; *COPD* chronic obstructive pulmonary disease; *Hb* hemoglobin; *ALB* albumin; *ASA* American Society of Anesthesiologists; *TXA* tranexamic acid. *: *p* < 0.05

**Fig. 1:**
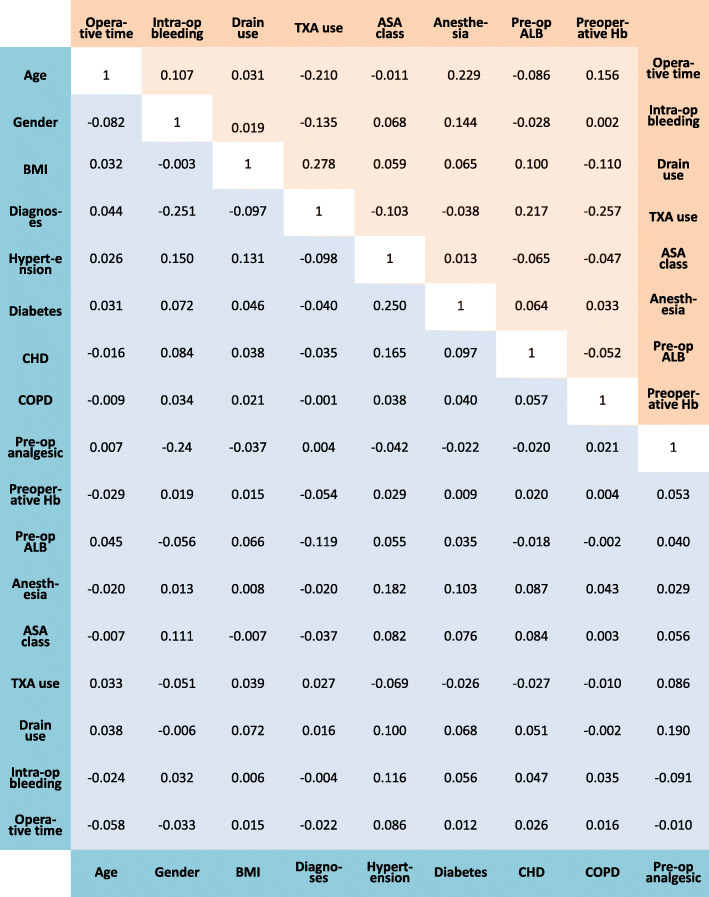
Spearman’s rank correlations between covariates enrolled in logistic and linear regression analyses. BMI: body mass index; OA: osteoarthritis; CHD: coronary heart disease; COPD: chronic obstructive pulmonary disease; Hb: hemoglobin; ALB: albumin; ASA: American Society of Anesthesiologists; TXA: tranexamic acid; Pre-op: pre-operative; Intra-op: Intra-operative

### Statistical analyses

Continuous variables were summarized as mean ± standard deviation, and categorical variables were presented as frequency (proportion). The correlations of the covariates enrolled in logistic and linear regression analyses were quantified using Spearman’s rank correlation analysis. The crude model of transfusion rate and PLOS regarding the use of tourniquet in TKA were built using univariate analyses. Logistic and linear regression analyses were used to adjust other variables and explore the relationships between these variables. The relationship between PLOS and transfusion rate and the enrolled covariates was presented as relative risk (RR) and 95% confidence interval (CI), and partial regression coefficient and 95%CI, respectively. A value of *P* < 0.05 was considered to indicate statistical significance. All data analyses were performed using SPSS version 21 software (IBM, Armonk, NY, USA).

## Results

### Data completeness analyses and Spearman’s rank correlations between covariates

Based on comparison with data from the hospital information system, the completeness of reporting was nearly 94% for unilateral primary TKA. The portion of missing or incorrect information of enrolled variables was generally less than 0.5%. Spearman’s rank correlation analyses showed that the absolute value of rank correlation coefficient between covariates enrolled in logistic and linear regression analyses ranged from 0.002 to 0.278, which indicated that correlations between the included variables were low and met the requirements of logistic and linear regression analyses Fig. 1.

### Transfusion rate and PLOS for all patients and two groups

**Fig. 2:**
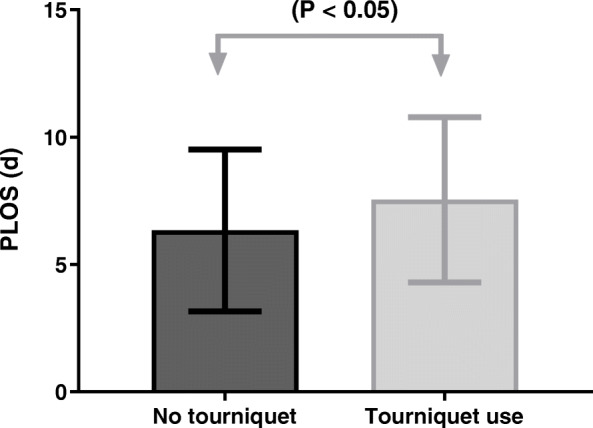
The postoperative length of stay of two groups

The total transfusion rate was 12.71% for all enrolled patients who underwent unilateral primary TKA, while 14.57% for the tourniquet group and 6.47% for the no-tourniquet group. The average PLOS was 7.41 ± 3.57 days for all patients after TKA, while 7.72 ± 3.54 days for the tourniquet group and 6.44 ± 3.48 days for the no-tourniquet group (Table 2 and Fig. 2).

**Table 2:** The transfusion rate and postoperative length of stay in routine primary TKA

Variables	No Tourniquet (*n* = 1423)	Tourniquet use (*n* = 4902)	All (*n* = 6325)
Transfusion N (%)	92 (6.47%)	712 (14.52%)	804 (12.71%)
PLOS (days)	6.44 ± 3.48	7.72 ± 3.54	7.41 ± 3.57

*PLOS* postoperative length of stay

### Impact of tourniquet use on transfusion rate and PLOS in unilateral primary TKA

As shown in Table 3, several statistical models including different covariates were built to evaluate the influence of tourniquet use in TKA on transfusion rate and PLOS. First, univariate analyses (crude model) showed that tourniquet use in TKA was associated with a higher transfusion rate (RR = 2.458, 95%CI 1.962–3.081, *P* < 0.001) and longer PLOS (*B* = 1.280, 95%CI 1.066–1.494, *P* < 0.001). After adjusting for age, sex, BMI, and orthopedic diagnoses using logistic and linear regression analyses, our results showed that tourniquet application remained correlated with a higher transfusion rate (RR = 2.604, 95%CI 2.075–3.267, *P* < 0.001) and longer PLOS (*B* = 1.294, 95%CI 1.080–1.508, *P* < 0.001) (model 1).

**Table 3:** The results of logistic and liner regression analyses in routine primary TKA

Variables	Transfusion rate		PLOS	
	RR (95%CI)	*P* value	B (95%CI)	*P* value
Crude model	2.458 (1.962–3.081)	0.000	1.280 (1.066–1.494)	0.000
Model 1	2.604 (2.075–3.267)	0.000	1.294 (1.080–1.508)	0.000
Model 2	2.319 (1.841–2.922)	0.000	1.278 (1.061–1.495)	0.000
Model 3	1.888 (1.449–2.461)	0.000	0.923 (0.690–1.156)	0.000

*PLOS* postoperative length of stay

Model 1: controlled for age, gender, body mass index and diagnoses

Model 2: controlled for model 1 + hypertension, type 2 diabetes, chronic obstructive pulmonary disease, coronary heart disease, preoperative analgesic use, hemoglobin and albumin

Model 3: controlled for model 2 + method of anesthesia, American Standards Association class, operative time, intraoperative bleeding, tranexamic acid and drain use

After further adjustment for preoperative comorbidities (hypertension, type 2 diabetes, coronary heart disease, chronic obstructive pulmonary disease), preoperative analgesic use, and preoperative hemoglobin and albumin based on model 1, using logistic and linear regression analyses tourniquet use was still correlated with a higher transfusion rate (RR = 2.319, 95%CI 1.841–2.922, *P* < 0.001) and longer PLOS (*B* = 1.278, 95%CI 1.061–1.495, *P* < 0.001) (model 2). Finally, After further controlling for all covariates (covariates in model 2 plus the operative variables including anesthesia method, ASA class, intraoperative blood loss, operative time, and TXA,and drain use), tourniquet use during unilateral primary TKA remained associated with a higher transfusion rate (RR = 1.888, 95%CI 1.449–2.461, *P* < 0.001) and longer PLOS (*B* = 0.923, 95%CI 0.690–1.156, *P* < 0.001), as presented in model 3.

## Discussion

By evaluating the data of 6325 patients, this study aimed to identify the impact of tourniquet use on transfusion rate and PLOS in patients undergoing primary unilateral TKA. Our findings suggested that tourniquet application during surgical procedure was correlated with a higher transfusion rate and a longer PLOS in patients who underwent unilateral primary TKA.

Although several studies have evaluated the influence of tourniquet use during TKA on blood loss and transfusion, no consensus has been reached about whether or not to use a tourniquet in routine primary TKA because of the diversity of current literature results [17]. A retrospective cohort study of 117 patients by Barros et al. [18] showed that tourniquet use in TKA resulted in a lower decrease in hemoglobin and hematocrit as well as fewer necessary blood transfusions. Surprisingly, Schnettler et al. [19] reported that tourniquet application during TKA resulted in a paradoxical increase in blood loss. Similarly, a meta-analysis of 13 randomized controlled trials (RCTs) involving 859 patients demonstrated that tourniquet use could minimize intraoperative blood loss and total blood loss, but increased postoperative total blood loss [20]. Schnettler et al. [19] suggested that increased blood loss from tourniquet use during TKA was likely the result of increased hidden blood loss (if a closed suction drain was not used), which accounts for more than half of the total blood loss, caused by the blood volume permeating through the adjacent soft tissues and joint space of the knee after the skin is closed following a surgical procedure [21]. The reason for this may be that tourniquet application augments fibrinolytic activity, which leads to increased hidden blood loss after tourniquet release [22].

For many orthopedists, reducing intraoperative blood loss, which potentially offers a relatively blood-free operating field and tight fixation of bone cement, is a crucial reason for using a pneumatic tourniquet during TKA. However, the controlled hypotension technique, which allows TKA to proceed without a tourniquet, is increasingly being used in total joint arthroplasty (TJA) [23]. An RCT performed by Juelsgaard et al. [24] reported that controlled hypotension could not only minimize both intraoperative blood loss and TBL, but also reduce the need for blood transfusion without increasing the risk of cardiopulmonary, cerebral, or renal complications. Additionally, TXA has been more extensively used in recent years to inhibit the fibrinolytic system and reduce blood loss during TJA. An RCT performed by Huang et al. [14], which involved 150 patients who underwent TKA, revealed that tourniquet use results in no reduction in intraoperative and hidden blood loss beyond that provided by TXA alone; moreover, the patients treated with TXA without a tourniquet had better early clinical outcomes, including a lower ratio of postoperative knee swelling, less postoperative pain, and better early knee function, compared with those treated with a tourniquet during surgery [25].

Tourniquet application in TKA is associated with several adverse effects. Previous studies [9, 10] suggested that use of a tourniquet in TKA decreases the thigh and quadriceps muscle volumes and delays the postoperative recovery of knee function, which may be correlated with ischemia–reperfusion injury caused by a tourniquet. Several previous studies showed that ischemia–reperfusion injury in human skeletal muscle accompanied by the full release of oxygen free radicals and inflammatory cytokines could reduce protein synthesis and increase protein degradation by triggering cascades of cellular processes, which include inhibiting the protein synthesis pathway of cap-dependent translation initiation and elongation, and upregulating the ubiquitin proteasome system, one of the important pathways of skeletal muscle proteolysis [26–28]. Besides this, tourniquet use in TKA may be related to increased postoperative knee pain and swelling. A systematic review and meta-analysis of 46 RCTs demonstrated that tourniquet use in TKA caused more pain [29]. Moreover, two studies [30, 31] reported that tourniquet application in TKA was associated with a higher opioid consumption, which equates to increased pain related to tourniquet use. Liu et al. [32] demonstrated that tourniquet use in TKA hampers wound healing in the early postoperative period, indicating an association with hypoxia in the edges of the wound immediately postoperatively caused by tourniquet application. Furthermore, besides impairing the local muscle metabolism and wound healing, ischemia–reperfusion injury to the lower extremity caused by tourniquet use elevates local and systemic oxidative stress as well as inflammatory reactions and impairs renal function, especially for patients undergoing simultaneous bilateral TKA [26, 33].

PLOS or length of stay (LOS) is regarded as an important outcome and one of the non-substitutable markers of excellence when evaluating ERAS programs in TJA [34]. Shortening LOS/PLOS, which indicates higher hospital efficiency and productivity, is a significant target worldwide [35]. Our study revealed that tourniquet use in TKA was associated with longer PLOS, which may be caused by factors already mentioned such as compromised quadriceps muscle, increased postoperative pain and swelling, and increased postoperative blood loss. Similarly, several previous studies also showed that tourniquet application hampered functional recovery postoperatively, which also was associated with a longer PLOS [31, 36, 37].

There some limitations to this study. First, accurate pressure and duration of tourniquet application during surgical procedures was not recorded in detail in the database, and we were thus unable to evaluate the impact of different tourniquet pressures and duration times on transfusion rates and PLOS. Although some previous studies had investigated the efficiency and safety of different tourniquet pressures [38, 39] and duration times [40, 41] in TKA, we surmised that the disadvantages and risks associated with tourniquet use may outweigh its benefits. Second, the details of TXA and drain use were not recorded—only whether TXA and drain was used for each patient—and the schemes of TXA and drain use were different for every hospital participating in our study, which may be associated with blood loss and transfusion rates. Third, the details of the controlled hypotension technique were not recorded in our database, and the anticoagulation was not also analyzed due to the anticoagulant schemes of included patients have very large heterogeneity. Both these two variables could affect the blood loss and transfusion rates. Fourth, some important outcomes, such as the range of motion of the treated knee and the postoperative complications, were not evaluated. Even so, to our best knowledge this study includes the largest cohort of patients to date to provide conclusive results in regard to tourniquet application during TKA procedures and its impact on transfusion rate and PLOS. This study will be a useful reference for orthopedists in their decision-making process regarding routine tourniquet application in primary unilateral TKA.

## Conclusion

In sum, this real-world study suggests that tourniquet use in TKA is associated with a higher transfusion rate and a longer PLOS. The impact of tourniquet use on transfusion rate and PLOS should be taken into account in clinical practice.

## Data Availability

Please contact author for data requests.

## Abbreviations

ASA: American Society of Anesthesiologists
CI: confidence interval
ERAS: enhanced recovery after surgery
LOS: length of stay
PLOS: postoperative length of stay
RCTs: randomized controlled trials
RR: relative risk
TXA: tranexamic acid
TKA: total knee arthroplasty
TJA: total joint arthroplasty
